# The rarity of gene shuffling in conserved genes

**DOI:** 10.1186/gb-2005-6-6-r50

**Published:** 2005-05-09

**Authors:** Gavin C Conant, Andreas Wagner

**Affiliations:** 1 Department of Genetics, Smurfit Institute, University of Dublin, Trinity College, Dublin 2, Ireland; 2 Department of Biology, The University of New Mexico, Albuquerque, NM 87131-0001, USA

## Abstract

**Background:**

Among three sources of evolutionary innovation in gene function - point mutations, gene duplications, and gene shuffling (recombination between dissimilar genes) - gene shuffling is the most potent one. However, surprisingly little is known about its incidence on a genome-wide scale.

**Results:**

We have studied shuffling in genes that are conserved between distantly related species. Specifically, we estimated the incidence of gene shuffling in ten organisms from the three domains of life: eukaryotes, eubacteria, and archaea, considering only genes showing significant sequence similarity in pairwise genome comparisons. We found that successful gene shuffling is very rare among such conserved genes. For example, we could detect only 48 successful gene-shuffling events in the genome of the fruit fly *Drosophila melanogaster *which have occurred since its common ancestor with the worm *Caenorhabditis elegans *more than half a billion years ago.

**Conclusion:**

The incidence of gene shuffling is roughly an order of magnitude smaller than the incidence of single-gene duplication in eukaryotes, but it can approach or even exceed the gene-duplication rate in prokaryotes. If true in general, this pattern suggests that gene shuffling may not be a major force in reshaping the core genomes of eukaryotes. Our results also cast doubt on the notion that introns facilitate gene shuffling, both because prokaryotes show an appreciable incidence of gene shuffling despite their lack of introns and because we find no statistical association between exon-intron boundaries and recombined domains in the two multicellular genomes we studied.

## Abstract 2

The incidence of gene shuffling is estimated in conserved genes in 10 organisms from the three domains of life. Successful gene shuffling is found to be very rare among such conserved genes. This suggests that gene shuffling may not be a major force in reshaping the core genomes of eukaryotes.

## Background

How do genes with new functions originate? This remains one of the most intriguing open questions in evolutionary genetics. Three principal mechanisms can create genes of novel function: point mutations and small insertions or deletions in existing genes; duplication of entire genes or domains within genes, in combination with mutations that cause functional divergence of the duplicates [1-3]; and recombination between dissimilar genes to create new recombinant genes (see, for example [4,5]). We here choose to call only this kind of recombination gene shuffling, excluding, for example, duplication of domains within a gene. In such a gene shuffling event, the parental genes may be either destroyed or preserved [6]. Gene shuffling is clearly the most potent of the three causes of functional innovation because it can generate new genes with a structure drastically different from that of either parental gene. Laboratory evolution studies show that gene shuffling allows new gene functions to arise at rates of orders of magnitudes higher than point mutations [7,8].

**Figure 1:**
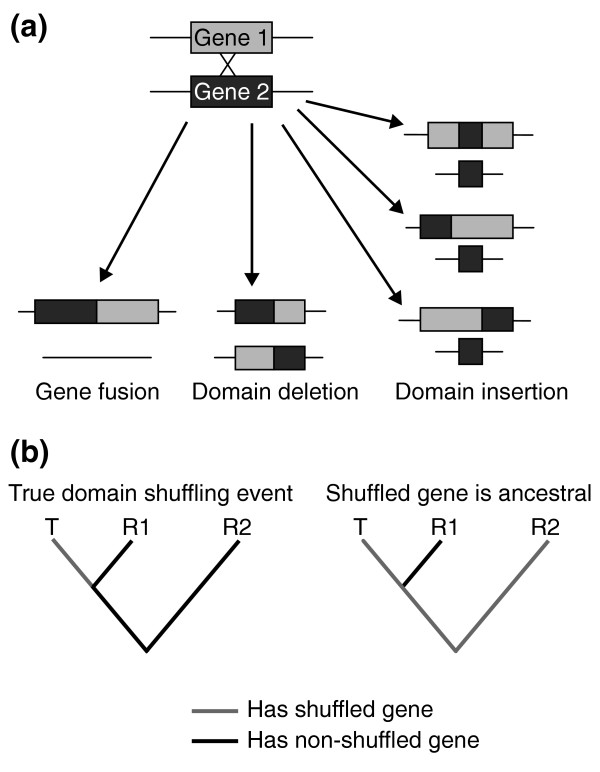
Identifying gene shuffling. **(a) **Gene shuffling and how it changes gene structure. The three scenarios of 'domain insertion' represent insertions of domains from gene 2 into gene 1. The reciprocal insertions (gene 1 into gene 2) are not shown. **(b) **Distinguishing true from spurious recombination events. In a spurious recombination event, reference genome R1 has two separate genes, where both T and R2 have a single, shuffled gene. The most parsimonious explanation for this observation is that the shuffled gene was present in R1 but was lost since R1's divergence from T.

Much is known about rates of point mutations [9] and of gene duplications [10,11]. In contrast, the rate at which gene shuffling occurs is relatively unexplored, despite the importance of shuffling for functional innovation. To be sure, anecdotal evidence suggests that successful gene shuffling occurs and that it creates genes with new functions [4]. In particular, proteins are often mosaics of domains that are characterized by sequence and structural similarity [12-19]. Many domains occur in multiple proteins of different functions, suggesting that new proteins can arise through the combination of domains of other proteins, a process requiring recombination. In addition, many studies have systematically identified one subclass of gene-recombination events - gene fusions [20-24]. These studies count gene fusion events in a genome of interest relative to multiple, often very distantly related, species. Because fused genes often have similar functions, identification of fusion events can aid in inferring gene functions. Here we address a question that goes beyond the above studies: how frequent is gene shuffling in comparison with other forces of genome change, such as gene duplication? This problem is difficult because of the many possible outcomes of recombination events. These outcomes fall into three principal categories, gene fusions, domain deletions, and domain insertions (Figure 1a). To identify these outcomes systematically on a genomic scale is computationally intensive, which has limited our analyses to a modest number of genomes (Table 1).

One can identify gene-shuffling events either from protein sequence information or from information about protein structure. Structure-based approaches [12-15] have the advantage of being able to detect recombination events where sequence similarity between a recombination product and its parents has eroded beyond recognition. However, because two very distantly related structural domains can also have arisen through convergent evolution [25,26], identifying common ancestry of two domains based on structure alone can be problematic. As a further limitation, structure-based approaches can only identify recombination events that respect the boundaries of protein domains, whereas some successful recombination events may occur within domains [27-29]. In addition, structural information is not available for all genes. For example, the Pfam database of protein domains [30] contains no structural information for more than 40% of proteins in budding yeast (*Saccharomyces cerevisiae*). Structure-based approaches may thus miss many shuffled genes. Because of these issues we chose a sequence-based approach which allows us to search for shuffling events without making restrictive assumptions regarding their nature. Essentially, our search imposes no restrictions on shuffling except that it must merge in a single gene two protein-coding sequences that were previously a part of two different genes. We thus avoid assuming that shuffling occurs only at domain boundaries or with certain recombination mechanisms without precluding either possibility. Our analysis can also account for gene-duplication events in either parental or recombined genes.

We here identify gene-shuffling events that have occurred in a 'test' species T since its divergence from a reference species R1. A gene in the test genome whose parts match more than one gene in the reference genome is a candidate for a gene-shuffling event that has occurred since the common ancestor of the two genomes. Our analysis also uses a third genome (reference genome R2) to prevent gene fission or gene loss in the reference genome R1 from resulting in spurious identification of gene shuffling events. Because R2 is an outgroup relative to T and R1, it allows us to detect such events in R1 (see Figure 1b). Like any comparative sequence-based approach, our analysis depends on detectable sequence similarity among genes. In other words, our analysis excludes rapidly evolving genes.

## Results

### Little gene shuffling in closely related genomes

Mosaic proteins are not rare in most genomes, which suggests that successful gene shuffling might be frequent on an evolutionary timescale. We thus searched four closely related genomes for shuffled genes. These genomes fit three essential criteria for this analysis: close taxonomic spacing; availability of complete genome sequence; and, most important, reliable gene identification. (Gene identification is notoriously unreliable in higher organisms because of their complex gene structure.) These species were the four yeasts *Saccharomyces cerevisiae*, *S. paradoxus, S. bayanus *and *S. mikatae *[31], which diverged from their common ancestor between 5 and 20 million years ago (Mya) [31]. We found multiple candidate genes for shuffling in different T-R1 pairs of these four species. However, almost all of these candidates proved spurious for a variety of reasons: First, some of them occurred in two or more species in a manner inconsistent with these species' phylogeny, or they matched more closely a single reference species gene than their two putative parents. Both observations make recent recombination an unparsimonious explanation for a gene's origin (Figure 1b). Second, the putative shuffled domains in some candidate genes had a synonymous, or silent, nucleotide divergence from their parental domains that differed by a factor of two or more. However, the recombined parts of a shuffled gene should show equal sequence divergence to their respective parental genes, because they have identical divergence times (namely the time since T and R1 shared a common ancestor). We used silent nucleotide substitutions as an indicator of sequence divergence because such substitutions are under little or no selection and thus accumulate in an approximately clock-like fashion [32]. Use of amino-acid changing (nonsynonymous) substitutions (*K*_a_) as an indicator led to similar conclusions. After exclusion of all such spurious genes, only two potential shuffled genes remained in our analysis, which indicates a low incidence of gene shuffling.

### Shuffled genes in distantly related genomes

Because our analysis of yeast genomes suggests that gene shuffling may be rarer than one might expect, the need arises to study more distantly related genomes. This raises two principal problems. First, such an analysis will miss events where either parental or shuffled genes have diverged beyond sequence recognition since two genomes shared a common ancestor. We thus emphasize that our analysis applies only to 'core' genomes: genes so well conserved that their homology even among distantly related species is beyond doubt. The incidence of shuffling among more rapidly evolving genes may be different and cannot be estimated with this approach. In this regard, we also note that our analysis cannot simply use multiple outgroups for a given test genome [20-24] to solve this problem, because doing so has the potential to misestimate shuffling rates by making wrong assumptions about the most parsimonious placement of such events (especially among prokaryotes, where horizontal transfer of shuffled genes may occur). For the remainder of our analysis, we chose ten distantly related genomes (Table 1) that best met the joint requirements of well known phylogenetic relationships and reliably annotated genome sequences (which is often problematic for the higher eukaryotes).

In addition to raising problems, the comparison of distantly related genomes also has one advantage: such genomes are more likely to be annotated independently from each other than are closely related genomes. In a group of closely related genomes, the first sequenced genome may often be used as a guidepost to annotate the other genomes, which may lead to errors (for instance, by misidentifying a shuffled region as an intron).

The number of shuffled genes we found is modest even for anciently diverged species pairs. For example, only 82 gene-shuffling events among the 5,800 genes considered (Table 2) may have been preserved in *Caenorhabditis elegans *since its common ancestor with *Drosophila melanogaster*, which lived around 600 Mya [33]. Similarly, only four surviving recombination events (out of 2,300 genes) may have occurred in the budding yeast *Saccharomyces cerevisiae *since its split from the fission yeast *Schizosaccharomyces pombe *more than 300 Mya [34]. We emphasize that all these numbers refer to shuffled genes that have 'survived': extant genome sequences alone are insufficient for estimating the frequency of the recombination events themselves, since the products of these events often will not become fixed in populations.

One further observation indicates the rarity of gene shuffling: most shuffled genes contain at least one domain of low sequence similarity to a parental gene. The above analysis is based on identifying sequence domains as homologous in a parental and recombined gene if they show more than 35% amino-acid sequence identity. Increasing this identity threshold to 40% can reduce the number of candidate shuffled genes dramatically (see Table 2). For instance, it removes 28 of 48 shuffled fruit fly genes and half of the shuffled fission yeast genes. This observation underscores that shuffling is rare among highly conserved genes: otherwise we would see higher sequence similarities among parental/recombined domain pairs.

**Figure 2:**
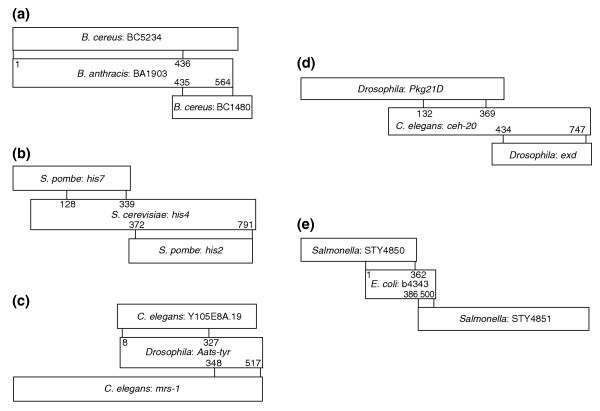
Representative examples of shuffled genes identified. **(a) ***Bacillus anthracis *M23/M37 peptidase BA1903, the result of a domain exchange between *B. cereus *genes BC5234 (12098), a *N*-acetylmuramoyl-L-alanine amidase and BC1480(08460.1), another M23/M37 peptidase. **(b) **A fusion of the fission yeast genes *his7 *(a phosphoribosyl-AMP cyclohydrolase) and *his2 *(a histidinol dehydrogenase) to produce the budding yeast *his4 *gene, which is involved in histidine biosynthesis. The budding yeast gene appears to combine the functions of the two fission yeast genes [35]. **(c) **The fruit fly gene *Aats-tyr *is a tyrosyl-tRNA synthetase (Flybase annotation) [36]. It is a probable recombination product of a predicted worm methionyl-tRNA synthetase gene *mrs-1 *(WormBase annotation) [37] and a second worm gene *Y105E8A.19 *of unknown function. **(d) ***C. elegans *gene *ceh-20*, which encodes a homeodomain protein. This gene appears to be the result of a domain exchange between the *Drosophila *genes *exd *(*extradenticle*, also a homeodomain protein) and *Pkg21D *(cGMP-dependant protein kinase). **(e) ***E. coli *b4343, a hypothetical protein apparently formed via a domain exchange between *Salmonella *genes STY4850 (annotated as a DEAD-box helicase-related protein) and STY4851 (hypothetical protein). The numbers in the recombinant gene box are amino-acid positions in the protein product, indicating the portion of the protein derived from each of its 'parental' proteins.

Figure 2 shows representative examples of shuffled genes, illustrating some of the types of recombination diagrammed in Figure 1a. For example, Figure 2b shows the budding yeast *his4 *gene, which is involved in histidine biosynthesis. This (apparent fusion) gene appears to combine the functions of the two fission yeast genes *his7* (a phosphoribosyl-AMP cyclohydrolase) and *his2 *(a histidinol dehydrogenase) [35]. Figure 2c shows the fruit fly gene *Aats-tyr*, a tyrosyl-tRNA synthetase [36]. This gene is a likely recombination product of a predicted worm methionyl-tRNA synthetase gene *mrs-1 *[37] and a second worm gene *Y105E8A.19 *of unknown function. A list of all shuffled genes identified in these ten genomes is available in Additional data file 1.

### Gene shuffling and structural domains

**Figure 3:**
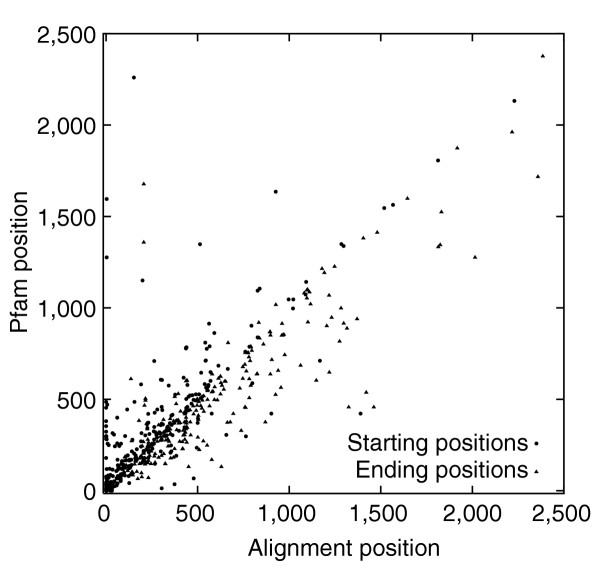
Association between recombined sequence domains and Pfam structural domains. The horizontal axis shows the starting and ending positions of the sequence domains in recombined genes (in amino acids, relative to the translation start site of the gene). The vertical axis shows the starting and ending positions of the Pfam domain closest to each recombined sequence domain.

Because our approach is based on sequence domains, we wished to find out whether the recombined regions of shuffled genes match structural protein domains. If so, this would indicate that successful recombination events - events preserved in the evolutionary record - occur mostly at structural domain boundaries. To address this question, we used the Pfam database [30,38] of protein domains to identify domains in our shuffled genes that were significant at E ≤ 10^-5^. These Pfam domains were compared to the sequence alignments that we used to identify shuffled genes in the first place. As Figure 3 shows, the boundaries of recombined sequence domains and Pfam structural domains tend to coincide (*P *< 0.001 using a domain randomization approach, see Materials and methods). However, Figure 3 also suggests that not all successful recombination events occur at structural domain boundaries. Experimental and computational work on individual proteins [27] supports the notion that successful recombination occurs preferentially, but not exclusively, at structural domain boundaries.

### Gene shuffling and exon-intron structure

The exon-shuffling/introns-early hypothesis [39-41] predicts that exon-intron boundaries delimit functional domains and hence that recombination events that preserve exons would be more likely to yield functional recombinant proteins. Long introns also increase the probability of a DNA-level recombination event preserving exons (since in this case the number of possible DNA-level recombination events leading to the same recombinant protein may be quite large), a further reason to expect an association of shuffling boundaries and exon boundaries. The two multicellular eukaryotes (*Drosophila *and *C. elegans*) have a sufficient number of introns to allow us to test this prediction by comparing the boundaries of recombined sequence domains to the positions of introns in the sequences in question. However, contrary to these expectations, we found no tendency for our shuffling boundaries to associate with exon-intron boundaries (*P *> 0.1, domain randomization test; see Materials and methods).

### The incidence of gene shuffling

We cannot estimate the incidence of gene shuffling in absolute (geological) time, because divergence dates for most of our test species are unknown or highly uncertain. In addition, the rarity of gene-shuffling events further complicates such estimates. However, we can obtain order-of-magnitude estimates of the incidence of gene shuffling relative to the incidence of other mutational events important in genome evolution. One such event is gene duplication, whose incidence has been estimated previously [10,11].

To compare the incidence of gene duplication to that of gene shuffling, we cannot rely on the silent nucleotide divergence among duplicate genes to estimate the rate of duplication, as is commonly done [10,11], because several of our study genomes are very distantly related. We thus estimated the rate at which gene duplications occurred in a test species T since its common ancestor with R1 using the following approach. We identified, for each test species T, all genes that had only a single homolog in the reference species R1. We denote the number of these reference species genes as *g*. Second, for each of these genes *i *we determined the number *n*_*i *_of test species genes homologous to gene *i*. If this number *n*_*i *_is greater than 1, then the test species homolog of gene *i *underwent one or more duplications since the common ancestor of T and R1. We estimated the (minimal) number of duplication events necessary to establish a gene family of size *n*_*i *_as . The total estimated number *d *of gene duplications for the *g *reference species genes then calculates as the sum . Values for *g *and *d *are shown for each reference species in Table 2. One can view the ratio *d/g *as the per-gene incidence of gene duplication.

**Figure 4:**
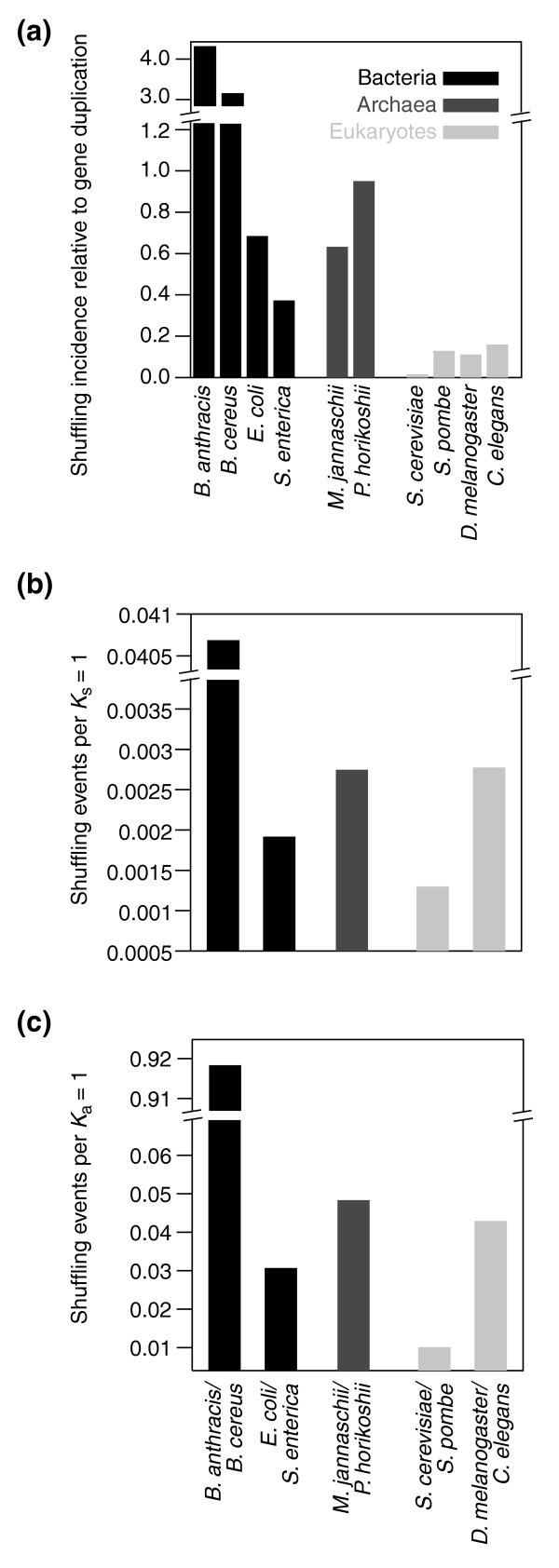
Incidence of gene shuffling relative to various other mutational events. **(a) **Gene duplication, **(b) **silent nucleotide substitutions, and **(c) **amino-acid changing nucleotide substitutions for the species pairs indicated on the horizontal axis. Note the scale breaks on the vertical axes.

We then used this ratio to estimate the ratio of gene-shuffling events per gene duplication event (Table 1). To do so, we first had to estimate the number of gene-shuffling events per gene, which we obtained by dividing the number *s *of gene-shuffling events in a test species T (Table 1) by the average number *h *of genes in T or R1 with detectable sequence similarity to genes in the other genome (Table 2). This approach of estimating the number of gene-shuffling events per gene compensates for the reduced ability to recognize gene homology in distantly related genomes. The ratio of shuffling events per duplication can then be calculated as (*s*/*h*)/(*d*/*g*). Figure 4a compares this ratio for the organisms we studied. The bacteria analyzed share with the archaeans a high incidence of gene shuffling relative to duplication, while the eukaryotes show a much lower incidence. The *Bacillus *species (*B. anthracis *and *B. cereus*) have a much higher relative incidence of gene shuffling than any other species pair we studied.

Other mutations useful to calibrate the incidence of gene shuffling are nonsynonymous (amino-acid replacement) and synonymous (silent) mutations on DNA. Synonymous substitutions are an indicator of divergence time between two genes or species because they are subject to few evolutionary constraints and thus may change at an approximately constant (neutral) rate [32]. We estimated the incidence of gene shuffling relative to synonymous substitutions by first determining the average fraction, *K*_s_, of synonymous nucleotide changes per synonymous nucleotide site for 100 orthologous genes in a T-R1 species pair. We then simply divided the number of gene-shuffling events per gene (*s/h*) by this average *K*_s _(Figure 4b). The evolutionary distance of two of our species pairs (*E. coli *vs *Salmonella *and *B. anthracis *vs *B. cereus *was sufficiently low to allow us to directly calculate the average synonymous divergence for 100 pairs of randomly selected single-copy orthologs in the test and reference species (see Materials and methods). For the other species pairs, most synonymous sites are saturated with substitutions [32]. In these cases, we thus extrapolated the value of *K*_s _between R1 and T from that observed between either of these species and a third, closely related species (see Materials and methods for details). We emphasize that this procedure would be unsuitable to make evolutionary inferences for any one gene, because it introduces considerable uncertainty into our estimates. It is, however, adequate to identify the approximate, genome-wide patterns we are concerned with.

Finally, we also estimated, completely analogously, the number of gene-shuffling events per unit amino-acid divergence (*K*_a _= 1). These results are summarized in Table 1 and Figure 4c. The incidence of gene shuffling relative to silent and amino-acid divergence varies less systematically among the domains of life than that of gene shuffling relative to duplication. However, it is again apparent from these analyses that successful gene shuffling is very rare for conserved genes. For some species, crude estimates of the absolute geological time needed for two sequences to accumulate a pairwise divergence of one silent nucleotide substitution per silent site are available. In the fruit fly this amount of time is approximately equal to 64 million years [32]. During this period of time, we would expect only 5,864 × 2.8 × 10^-3 ^= 16 gene shuffling events to occur (Tables 1 and 2; 5,864 is the average number of fruit fly and worm genes in our core gene set). By way of comparison, even using our very conservative method of counting duplicate genes, we would expect 146 gene duplications in this period. Similarly, in the yeast *S. cerevisiae*, where *K*_s _= 1 synonymous substitutions accumulate every 100 million years [42], one would expect three shuffling events during this period of time (2,365 × 1.3 × 10^-3^), as compared to 200 gene duplications. We emphasize that these are order-of-magnitude estimates that mainly serve to underscore the rarity of successful gene shuffling.

A multidomain protein may include both distinct and repeated structural domains [13]. Multidomain proteins with repeated domains raise a special problem for identifying gene-shuffling events: a shuffling event followed by domain duplication might lead us to miss a shuffled gene because our local alignments of that gene to its parental genes would only include one copy of the duplicated domain and hence might reveal less than the 50% of alignable amino-acid residues we require (see Materials and methods). To assess whether this problem would substantially bias our results, we examined candidate shuffled genes that had been excluded by our criterion (that is, those having between 10% and 50% of their residues alignable). We asked whether a failure to account for domain duplication was responsible for their exclusion. After adding potentially duplicated domains to the aligned regions of these genes, we found that only a handful of them (two genes in *Drosophila *and three in *C. elegans*) met our 50% alignability threshold. Failure to account for domain duplications internal to a gene is thus not the reason for our low estimates of the incidence of gene shuffling.

Several lines of evidence show that successful gene shuffling is very rare for genes conserved between the distantly related genomes we studied. For the single-celled yeasts - currently the only group of very closely related eukaryotes with sufficiently reliable genome annotation - shuffling appears rare in the genome as a whole. In most of the genomes we analyzed, gene shuffling is much rarer than other important kinds of mutations affecting gene structure, such as gene duplication. For example, in the time that it takes to accumulate *K*_s _= 0.01 synonymous substitutions per synonymous site, other research indicates that ten fruit fly genes and 164 worm genes undergo duplication [10]. In contrast, each lineage has only a 50% chance of undergoing a successful gene shuffling event in the same amount of time (if one assumes our estimates can be applied to the entire genome). We note that our estimates of duplication rates are more conservative than those of others [10], partly because we limit ourselves to single-gene duplications. The fact that we still see a lower incidence of shuffling than duplication (Figure 4) is thus all the more remarkable.

## Discussion

The rarity of gene shuffling relative to gene duplication has a simple potential explanation. A gene duplication creates a copy of a gene while preserving an original that is able to exercise its function. In contrast, unless a recombination event is accompanied by gene duplication, the original (parental) genes disappear in the event. An organism may survive a recombination event only if neither parental gene was essential to its survival and reproduction or if the recombinant gene(s) can carry out the function of both parental genes. This rarity of successful gene shuffling stands in stark contrast to the frequency of DNA recombination itself, which is a ubiquitous process accompanying DNA replication and repair. This suggests that the vast majority of recombined genes have deleterious effects on the organism, which may be particularly true for the highly conserved genes examined here.

We emphasize that the rarity of gene shuffling we find is not in contradiction with earlier studies that have identified multiple gene fusions - a special, simple case of gene shuffling - in fully sequenced genomes [20-24]. These studies identified prokaryotic gene fusion events in one test genome relative to multiple, often very distantly related, reference genomes. Any such approach may find many fusion events even if such events are rare. Our data also do not rule out the possibility that shuffling played an important role in forming the conserved eukaryotic core genome, because the pertinent gene-shuffling events would have occurred before the divergence of the eukaryotic species pairs we examined. (The identification of such ancient shuffling events may require an approach based on protein structure.)

Furthermore, our results are not in contradiction with anecdotal evidence for the abundance of gene-shuffling events in some functional categories of genes [32]. The reason is that our results pertain to the average incidence of gene shuffling among conserved genes. Some genes may be shuffled at a much greater rate. Indeed, structural studies of multidomain proteins tend to find a few domains which co-occur with a wide variety of other domains (indicating the common shuffling of such promiscuous domains), whereas many other domains co-occur with only one or a few other domains (rare shuffling) [43]. Similarly, a lack of reliable genome annotation made it impossible to reliably identify gene-shuffling events in vertebrate genomes, where gene shuffling may be more frequent overall [44].

Perhaps the central caveat to our results regards sources of ascertainment bias. The comparison of distantly related genomes alone introduces a powerful source of ascertainment bias: we can only analyze gene-shuffling events for genes that have been sufficiently conserved to be recognizable in both genomes. However, shuffling might be more common among rapidly evolving genes. An additional possible source of bias is that after a successful gene-shuffling event the rate of amino-acid substitutions may be elevated as a result of directional selection on the newly created gene. Such a bias would cause us to underestimate shuffling frequencies in distantly related species even for conserved genes. Nonetheless, our results from the four closely related genomes argue against such a bias, because shuffling also appears rare in these genomes.

Another caveat is that our ability to identify successful gene-shuffling events depends on the continued presence of both parental genes in the reference genome. Genomes, however, occasionally lose genes. For instance, recent work has suggested that *S. cerevisiae *has lost roughly 10% of its genes since its last common ancestor with *S. pombe *[45]. If gene loss in other organisms occurs at comparable rates, our approach may slightly underestimate the number of recombination events in a lineage. However, note that gene loss affects our estimates of gene shuffling and gene duplication in similar ways, thus compensating for any such bias.

We used a second reference genome R2 to be able to exclude gene-fission events in reference genome R1. Such events can lead to misidentification of recombination in the test species and have been documented in several organisms [20]. Unfortunately, this approach fails if the same recombination event occurred twice, once in the lineage leading to reference species R2 and once in the lineage leading to test species T. Such a case of parallel evolution or homoplasy would lead us to misidentify a recombination event in T as a gene-fission event in R1. However, because successful gene shuffling is very rare in general, and because the required recombination event would have to occur at exactly the same position twice, this possibility is probably not a major confounding factor in our analysis.

A fourth caveat lies in the possibility that some of our recombinant genes may result from two independent recombination events. Our algorithm can identify such genes, but given the high sequence divergence of recombinant domains it may often be impossible to resolve the order of the individual recombination events. The generally small number of recombinant proteins implies that genes produced by two or more recombination events would be extremely rare. Indeed, among 203 identified recombinant genes, a mere 16 show matches to more than two parental genes, making these the only cases with indications that more than one recombination process was involved in their creation.

Finally, our approach to estimating the rate of gene duplication identifies only duplications of single-copy genes in the reference species. Multicopy genes may undergo duplication more frequently. We may thus have underestimated the number of gene duplications. As a result, the incidence of gene shuffling relative to gene duplication may be even lower than indicated by our estimates.

### Recombination and introns

Our findings speak to a long-standing debate in molecular evolution, a debate that revolves around the origin of introns. Introns are stretches of DNA that do not code for proteins and that separate exons, the protein-coding regions of genes. According to one point of view, introns originated early in the evolution of life, perhaps as early as the common ancestor of prokaryotes and eukaryotes [39-41]. According to this perspective, introns may have acted as spacers between exons and thus greatly facilitated recombination among exons to create new proteins. The opposite point of view is that introns arose late in life's evolution, perhaps as late as eukaryotes themselves [28,29] and thus had no role in gene shuffling earlier in life's history. Genes in two of our test genomes have a sufficient number of introns to test the hypothesis that introns facilitate gene shuffling. Neither of these genomes showed an association between gene-shuffling boundaries and exon position. In addition, neither of these genomes showed an elevated incidence of gene shuffling. Although based on a small number of genomes, this finding casts doubt on the importance of introns for gene shuffling, and it suggests that other aspects of genome architecture may be more important. One potential example is the organization of functionally related prokaryotic genes into operons. The close proximity of such genes may facilitate their reorganization and the generation of new functions, whether through simple fusion or fission or through more radical change.

### Natural selection or drift?

A nonhomologous recombination event that gives rise to a shuffled gene occurs in only one individual of a potentially large population. Does a shuffled gene typically rise to high frequency and become fixed through natural selection or genetic drift? To answer this question, one could in principle study the relationship between the rate at which fixed shuffled genes arise and population size (taking account of differences in nonhomologous recombination rates among species). Three possibilities exist in principle. First, there may be no relation between population size and the rate at which fixed shuffled genes arise. This would be the case if most gene-shuffling events are strictly neutral [46] or if they have very large beneficial effects. Second, there may be a positive relation between the rate at which fixed shuffled genes arise and population size. This would be the case if most shuffling events are mildly beneficial. The reason is that in this case selection favoring the fixation of a shuffled gene has to overcome the effects of genetic drift, which are weakest in large populations. Finally and perhaps most likely, there may be a negative association between population size and the rate at which fixed shuffled genes arise. This would be the case if most shuffling events are mildly deleterious. Such a negative association has been observed for several indicators of genome structure such as genome size, transposable element load, and rates of preservation of duplicated material [47].

Unfortunately, insufficient data are available to distinguish rigorously between these possibilities. First, estimates of effective population sizes *N*_e_, based on estimates of N_e_μ [47] and the mutation rate μ [9], exist only for three of our five pairs of genomes (*E. coli-Salmonella, S. cerevisiae-S. pombe *and *D. melanogaster-C. elegans*). Second, we have insufficient information on recombination rates (whose variation among genomes needs to be taken into account). Specifically, although estimates of homologous recombination rates are available for a few of our organisms [48-51], gene shuffling occurs strictly by nonhomologous recombination, whose rate need not have a simple relationship with the homologous recombination rate. A third difficulty is that recombination rates and mutation rates are conventionally measured per cycle of DNA replication, whereas we would require per-year estimates as well as estimates of absolute divergence times between our taxa of interest to make appropriate comparisons.

Despite such insufficient data, we can make the qualitative observation that the observed incidence of shuffling does not follow a simple pattern: For example, *S. cerevisiae *has a relatively high effective population size (N_e_μ is approximately half of that for *E. coli *[47] while μ is actually higher than that of *E. coli *[9]) and a high homologous recombination rate compared to *C. elegans *or *E. coli *[48-51], and yet it shows the lowest incident of gene shuffling of any of our taxa. In the slightly deleterious scenario above, we would instead expect yeast to show an incidence of shuffling greater than that of *E. coli*, while in the slightly beneficial scenario we would expect it to show an incidence greater than that of the multicellular eukaryotes.

A second qualitative observation is that the incidence of gene shuffling is not elevated in higher organisms relative to the rate of nucleotide substitutions. (The higher incidence of gene shuffling relative to gene duplication in prokaryotes from Figure 4a may be a consequence of the lower rate of gene duplication in these taxa.) This is consistent with the hypothesis that the fate of most shuffled genes is driven by natural selection rather than genetic drift. In other words, most shuffling events may not be neutral. This is again plausible if one considers that most gene-shuffling events change a gene's structure drastically. A corollary of this hypothesis is that preserved shuffled genes have been preserved for a reason - the benefit they confer to an organism. While rare in number, shuffled genes may thus be of great importance in organismal evolution.

Our analysis of gene shuffling has left many open questions, most notably about the association between the rate of sequence evolution and the rate of gene shuffling. To arrive at firm answers for this and other questions, we must be able to study shuffling rates not only for conserved proteins but also for rapidly evolving proteins. Such studies will require closely related genome sequences with reliable gene identification derived independently for each genome.

## Materials and methods

### Identifying shuffled genes

Our method identifies shuffled genes in a test genome (T) relative to a reference genome (R1). Table 1 shows the ten test genomes - two archaeal, six prokaryotic, and four eukaryotic genomes - we used in this analysis. Every pair of genomes R1-T occurs twice in Table 1, because one of two genomes can be used either as the test or the reference genome. To exclude spurious recombination events that reflect gene loss or fission in R1, the method also employs a second reference genome, R2. The two archaeans in our analysis were *Pyrococcus horikoshii *[52] and *Methanocaldococcus jannaschii *[53]. The R2 species for these archaeans was *Archaeoglobus fulgidus *[54]. The bacterial genomes we analyzed were those of *Escherichia coli *[55], *Salmonella enterica *[56], *Bacillus anthracis *[57], and *Bacillus cereus *[58]. The reference species R2 were *Bacillus subtilis *[59] for the *B. anthracis-B. cereus *comparison and *Haemophilus influenzae *[60] for the *E. coli*-*Salmonella *comparison. Our four eukaryotic genomes were budding yeast *Saccharomyces cerevisiae *[61], fission yeast *Schizosaccharomyces pombe *[62], nematode worm *Caenorhabditis elegans *[63] and fruit fly *Drosophila melanogaster *[64]. We used the genome of *Neurospora crassa *[65] as the R2 genome for all these eukaryotes.

To identify sequence homology between all genes in these genomes we used the Washington University implementation of gapped BLASTP [66,67], followed by exact pairwise local alignment using the Smith-Waterman algorithm [68] with a gap-opening penalty of 10 and a gap-extension penalty of 2, and the BLOSUM 62 scoring matrix [69]. We excluded from further analysis all gene pairs with BLAST E-values greater than 10^-6^, fewer than 50 aligned amino acids, amino-acid identity in the alignment of less than 35%, or alignments consisting of more than 50% low-complexity sequences as determined by the SEG program [70,71].

The requirement of 35% sequence identity may appear to bias our estimates of shuffling incidence between distantly related taxa. However, because we calculate these values relative to the total number of genes with the same (35%) degree of sequence identity between the test and reference genome (*h*), this bias is most likely to be small.

The result of this procedure is a list of partially or fully matching genes in the two species T and R1. We used this list to identify shuffled genes in the test genome T. Specifically, for each gene in the test genome T we searched for pairs of genes in the reference genome R1 that matched the test species gene, but in nonoverlapping or minimally overlapping regions. (To account for edge errors in local alignments, we allowed regions to overlap by a maximum of 20 residues). After having identified any such gene, we verified that it did not also have full-length homologs in the reference genome, because otherwise gene shuffling would not be the most parsimonious explanation of the gene's origin. We developed a special-purpose algorithm for this search [72], which identifies, for any one gene, the combination of local alignments to genes in the reference genome that covers the maximum number of residues in the shuffled gene. This algorithm can identify shuffled genes (genes to which two or more reference species genes contributed), but it will also return only a single alignment if this alignment is longer than any combination of non-overlapping alignments.

### Three criteria for validating shuffling events

We used three additional criteria to validate candidates for shuffled genes. First, we computed the proportion of a shuffled gene's amino-acid residues that could be aligned to its (parental) reference species genes. If this proportion is small, a gene may be too highly diverged for us to confidently ascertain that it is a recombination product. We excluded genes where this proportion was smaller than 50%. This requirement may appear restrictive, but additional analyses show that our conclusions hold even if it is completely eliminated. For example, eliminating this criterion increases the number of shuffled genes by a factor ranging from 1 (no increase, *E. coli*) to 4.2 (*C. elegans*), but the eukaryotes surveyed still show an incidence of shuffling smaller than the duplication rate, while the prokaryotes show similar frequencies of shuffling and duplication. We have maintained the 50% requirement throughout our main analysis to err on the side of caution: Putative shuffled genes with very short alignable regions to a parental gene are more likely to be false positives. They also do not belong in the set of genes conserved between T and R1, which is our focus here.

To motivate our second validation criterion, we note that in the eukaryotic test genomes some shuffled genes had undergone duplication. We identified gene duplicates as gene pairs with amino-acid divergences *K*_a_< 1 using a previously described and publicly available tool [73]. We counted each gene family of shuffled genes only once to avoid double-counting duplicates of shuffled genes.

**Figure 5:**
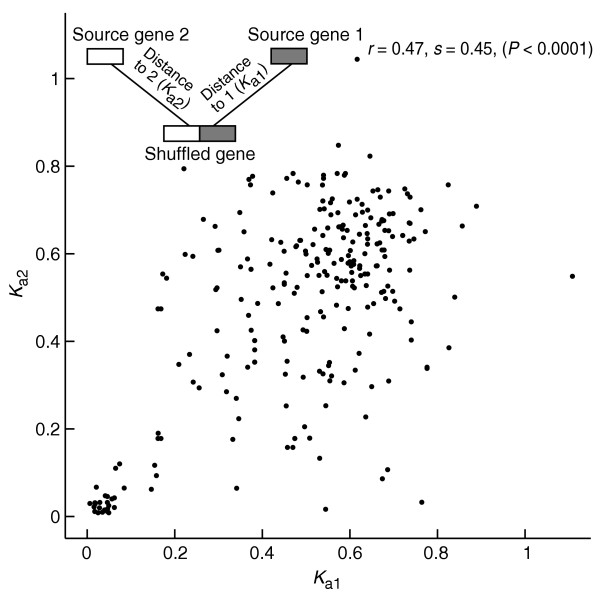
Similarity in sequence divergence between regions of shuffled genes. The amino-acid divergences (*K*_a_) of recombined domains to their respective parental counterparts are correlated. One outlying observation (*K*_a1 _= 3.26 and *K*_a2 _= 0.36) is not shown in this plot but was included in the calculation of correlation coefficients. Excluding this observation increases the Pearson correlation coefficient to 0.61 and leaves the Spearman correlation coefficient unchanged (*P *< 0.0001 for both).

A third indicator of true recombination is the divergence of different sequence domains within a putative shuffled gene. The recombined parts of a shuffled gene should show equal sequence divergence from their respective parental genes, if these parts have diverged in a clock-like fashion. The two principal indicators of DNA sequence divergence are the number of silent nucleotide substitutions at synonymous sites (*K*_s_) and the number of non-synonymous substitutions at amino-acid replacement sites (*K*_a_) [32]. We used the methods of Muse and Gaut and Goldman and Yang [74,75] to estimate these divergence indicators for our putative shuffled genes. Silent substitutions are subject to much weaker selection pressures than amino-acid replacement substitutions, and their rate of accumulation is thus more clock-like [32]. For our analysis of four closely related yeast species, we calculated the distance (*K*_a _or *K*_s_) between each sequence domain of a shuffled gene and its counterpart parental gene. We excluded candidate shuffled genes from further analysis if these distances (*K*_a _or *K*_s_) differed by more than a factor two between the different domains. For our highly divergent species (Table 1) we could not use synonymous divergence *K*_s _because most domain pairs had become saturated with silent substitutions. The (unavoidable) disadvantage of using only amino-acid divergence *K*_a _is that natural selection on amino-acid changes can cause non-clock-like evolution of domains. However, even with this caveat, our approach identifies true recombination events. Otherwise, our shuffled genes would not show the highly significant statistical association in amino-acid divergence *K*_a _we observe between sequence domain pairs (Figure 5; Pearson's *r *0.47, Spearman's *s *0.45, *P *< 0.0001 for both). Note that we did not exclude shuffled genes from our analysis of distantly related genomes based on unequal *K*_a _estimates.

### Detecting domain duplication in putative shuffled genes

Internal domain duplications could cause a shuffled gene to fail the first of the three test criteria above - 50% alignable residues with its parental genes - which would cause us to miss such genes. To assess how serious a problem such missed genes might be, we relaxed the first of the above test criteria, requiring only 10% alignable residues between a test gene and two or more potential parental genes. For test species genes that met this criterion, we then excised the portion of the test species gene that aligned to a reference gene, and recomputed a local alignment between this trimmed gene and the reference gene. If the test-species gene contained internal duplications of the reference gene, the trimmed sequence should still align to the reference gene. We added any new alignments found in this way to the original alignment combination and assessed whether the resulting combination of alignments met the required threshold of 50% alignable residues. For alignments that met our other prescreening criteria for candidate shuffled genes (50 amino acids in length and > 35% amino-acid identity) we iterated this excision procedure to determine the total number of repeated domains. This analysis identified only five additional shuffled genes (see above) and led us to conclude that internal duplications are not a major confounding factor in our analysis.

### Estimating relative frequencies of gene shuffling

We estimated the incidence of gene shuffling relative to two other frequent kinds of evolutionary events - gene duplications and nucleotide substitutions. To do so, we first needed to account for differences in genome size among our study species. We thus estimated the number of gene shuffling events per gene. This estimate poses a problem that stems from the different (and highly uncertain) times since common ancestry of our different T-R1 species pairs. The longer the time since common ancestry, the fewer genes (shuffled or not) with recognizable sequence similarity two species will share. Thus, when simply dividing the number of shuffled genes *s *by the total number of genes in a test genome, one may wrongly estimate the number of gene-shuffling events per gene. To account for this problem, we divided the total number of gene-shuffling events *s *by the number of recognizable homologs *h *shared between species T and R1 to obtain the number of gene shuffling events per gene, *s/h*. To obtain *h *itself, we determined the total number of genes in T with at least one homolog - using the criteria outlined earlier - in the genome of R1, the total number of genes in R1 with at least one homolog in T, and averaged these two numbers.

We then related this number of gene shuffling events per gene to the number of gene duplications in T since its common ancestor with R1. Because our test and reference genomes are only distantly related, we could not rely on the silent nucleotide divergence among duplicate genes to estimate the rate of duplication, as is commonly done [10,11]. We thus used the following, alternative, method. First, for each test species T, we identified all genes which match only a single gene in R1 at a BLAST threshold of 10^-6 ^and had 70% or more of their sequences aligned to that gene. The number of unique reference species genes matching one or more test species genes gives a baseline number *g *of genes before duplication. Second, for each such unique reference species gene *i *we identified duplicate pairs of test species genes that showed a pairwise amino-acid divergence *K*_a _which was less than either gene's amino-acid divergence from the putative ortholog *i*. For many genes *i*, more than one pair of test species genes fulfilled these criteria. Such genes represent families of *n*_*i *_duplicates of gene *i*. We estimated the (minimal) number of duplication events necessary to establish such a gene family of size *n*_*i *_as . The total estimated number *d *of gene duplications for the *g *reference species genes then calculates as the sum . Genes in very large families are more likely to undergo duplication than genes in smaller families [76]. For this reason, we excluded reference species genes *i *with more than ten duplicate genes from this analysis. Including such genes would tend to increase our estimated rate of gene duplication, meaning that the results shown in Figure 4a are conservative estimates of the excess of duplicates relative to shuffled genes. To estimate the rate of gene shuffling events relative to gene duplication events, we then divided the number of gene shuffling events per gene (*s/h*, obtained above) by the number *d/g *of gene duplication events per gene.

We also estimated the number of gene-shuffling events per unit of silent substitutions *K*_s _that accumulate in a gene. Two of our species pairs (*B. anthracis-B. cereus *and *E. coli-S. enterica*) allowed us to estimate synonymous divergence *K*_s _directly. For these two species pairs, we first identified 100 pairs of single-copy genes in each genome that are unambiguous orthologs [32,77]. We then divided the number of shuffled genes per gene by the average synonymous divergence *K*_s _of the orthologs with unsaturated synonymous divergence (> 97 for both species pairs) to obtain an estimate of the number of gene-shuffling events per unit change in K_s_.

For the three other genome pairs, this approach was not feasible because of their mostly saturated synonymous divergence. We thus had to estimate the average synonymous divergence K_s _between T and R1 by extrapolating from the synonymous divergence between T and a more closely related species. This approach relies on previous work [10] which indicates that the genome-wide average ratio *K*_a_/*K*_s _of amino-acid divergence to synonymous divergence approaches an asymptotic value for large numbers of distantly related genes. For each of the three T-R1 species pairs, our approach to estimating *K*_s _in this way consists of two steps. We first estimated the average amino-acid divergence K_a _between 100 randomly chosen unique single-copy orthologs of a T-R1 species pair. For the second step, we first identified an organism C with fully sequenced genome that is closely related to either T or R1. The genome of C should be sufficiently closely related to estimate *K*_s _reliably, but sufficiently distantly related to reliably estimate the asymptotic ratio of amino-acid to silent divergence. This organism was *Saccharomyces paradoxus *for *S. cerevisiae*; *Pyrococcus furiosus *[78] for *P. horikoshii*; and *Caenorhabditis briggsae *[79] for *C. elegans*. For each of these closely related genome pairs, we chose at random 100 single-copy gene pairs that were unambiguous orthologs. We calculated the average ratio *K*_ac_/*K*_sc _for these orthologs. We then used this value to extrapolate the average fraction *K*_s _of synonymous substitutions between genes in T and R1 as *K*_s _= *K*_a_/(*K*_ac_/*K*_sc_). This is the estimated average synonymous substitution rate between a T-R1 species pair. We then related the rate of gene shuffling to this extrapolation of *K*_s_. Specifically, we estimated the number of shuffling events per gene per one *K*_s _as (*s/h*)/(*K*_s_/2). (The reason for dividing the average *K*_s _by 2 is that our approach estimates the number of gene-shuffling events only for one of the two species of a T-R1 species pair.) We are well aware of the shortcomings of this approach, which averages heterogeneous substitution rates and assumes that the ratio of amino acid to silent divergence is constant within the taxonomic group considered. However, we emphasize that we use the approach here only to arrive at order-of-magnitude estimates of the incidence of gene shuffling. We also note that although we have not explicitly taken codon usage bias into account, the use of codon position-specific nucleotide frequencies (which partially account for such a bias, at a cost of larger estimate variances) increased all of our estimated average *K*_s _values without changing the patterns seen in Figure 4. Thus, the values in Figure 4 are conservative in the sense that the actual incidence of shuffling relative to *K*_s _may be lower than shown.

Third, and finally, we also estimated the number of gene-shuffling events per unit of amino-acid replacement substitutions *K*_a _that accumulate in a gene. To do so, we divided *s/h *as above by one-half the average amino-acid divergence (*K*_a_/2) of 100 unambiguous orthologs in a T-R1 species pair.

### Comparison of identified domains to Pfam database

Because our analysis was sequence and not structure-based, we used the Pfam database of structural protein domains [30,38] to evaluate how well the recombined sequence domains we identified matched structural domains. To do so, we queried all shuffled genes against the Pfam database and retained identified Pfam domains with E ≤ 10^-5^. We then compared the location of these structural domains to the location of the sequence domains in the shuffled genes. Specifically, we calculated the distance between each alignment domain and its closest Pfam domain. For the starting (*A*_s_) and ending positions (*A*_e_) of the alignment, and for the starting (*P*_s_) and ending positions (*P*_s_) of the Pfam domains found in the shuffled gene, we calculated the quantity . If *D *is very small, then the sequence domain and the Pfam structural domain overlap to a large extent. Parametric tests are not appropriate to evaluate the statistical significance of this association. We thus applied a gene-randomization procedure that created new alignment domains within each gene, domains whose starting and ending positions are uniformly distributed but cover the same proportion of the gene as did the original domains. Each randomized gene possessed the same number of simulated domains as observed domains, but with different positions and lengths. We then calculated *D *for the simulated alignment domains and compared its distribution to the empirically observed values of *D*. We applied an analogous approach to test whether exon/intron boundaries and shuffling boundaries are associated in our two multicellular eukaryotes (*C. elegans *and *Drosophila*). This approach substituted the closest exon for the closest Pfam domain and applied the same randomization procedure.

## Additional data files

Additional data file 1, available with the online version of this paper, contains a table listing all shuffled genes included in our analysis of the ten distantly related genomes.

## Supplementary Material

Additional File 1 A table listing all shuffled genes included in our analysis of the ten distantly related genomes. A table listing all shuffled genes included in our analysis of the ten distantly related genomes. Click here for file

## Figures and Tables

**Table 1:** Relative abundances of shuffled genes

Organism (T)	Reference taxa (R1)	Shuffled genes (*s*) (35%)	Shuffling events/duplication	Shuffling events/gene/*K*_s _unit	Shuffling events/gene/*K*_a _unit
*M. jannaschii*	*P. horikoshii*	7	0.63	2.7 × 10^-3^	4.8 × 10^-2^
*P. horikoshii*	*M. jannaschii*	7	0.95		
*B. anthracis*	*B. cereus*	21	4.33	4.1 × 10^-2^	0.92
*B. cereus*	*B. anthracis*	20	3.16		
*E. coli*	*S. enterica*	1	0.69	1.9 × 10^-3^	3.0 × 10^-2^
*S. enterica*	*E. coli*	5	0.37		
*S. cerevisiae*	*S. pombe*	4	0.015	1.3 × 10^-3^	1.0 × 10^-2^
*S. pombe*	*S. cerevisiae*	8	0.13		
*D. melanogaster*	*C. elegans*	48	0.11	2.8 × 10^-3^	4.3 × 10^-2^
*C. elegans*	*D. melanogaster*	82	0.16		

**Table 2:** Estimating the incidence of gene shuffling

Organism (T)	Reference taxa 1 (R1)	Reference taxa 2 (R2)	Shuffled genes (40%)	Sequences with detectable homology (*h*)	Number of duplicates/*R1 *genes tested	Duplication Rate (*d/g*)	Average *K*_s_*	Average *K*_a_
*M. jannaschii*	*P. horikoshii*	*A. fulgidus*	1	661	7/418	0.017	7.7	0.44
*P. horikoshii*	*M. jannaschii*	*A. fulgidus*	2	661	5/449	0.011	7.7	0.44
*B. anthracis*	*B. cereus*	*B. subtilis*	17	4,155.5	3/2568	0.0012	0.24	0.01
*B. cereus*	*B. anthracis*	*B. subtilis*	19	4,155.5	4/2624	0.0015	0.24	0.01
*E. coli*	*S. enterica*	*H. influenzae*	1	3,183.5	1/2182	0.0005	0.98	0.06
*S. enterica*	*E. coli*	*H. influenzae*	2	3,183.5	9/2140	0.0042	0.98	0.06
*S. cerevisiae*	*S. pombe*	*N. crassa*	3	2,365	104/946	0.110	3.9	0.50
*S. pombe*	*S. cerevisiae*	*N. crassa*	3	2,365	25/955	0.026	3.9	0.50
*D. melanogaster*	*C. elegans*	*N. crassa*	20	5,864	74/1008	0.073	8.0	0.52
*C. elegans*	*D. melanogaster*	*N. crassa*	34	5,864	98/1120	0.088	8.0	0.52

*Note that to obtain shuffling events/per gene/*K*_s _= 1.0 (Table 1) we divided the average *K*_s _by 2.

This was done because *K*_s _is a pairwise distance, meaning that it gives the sum of the divergences from the common ancestor to T and from the common ancestor to R1. The same was done for the *K*_a _analysis.
